# Knowledge and attitudes toward delirium: a multi-centered survey in a sample of physicians and pre-registration house officers in Egypt

**DOI:** 10.1007/s41999-025-01281-1

**Published:** 2025-07-30

**Authors:** Shaimaa Nabil Rohaiem, Maha Magdy Wahdan, Asmaa Fathy Abdellah Hassan

**Affiliations:** 1 Department of Geriatrics and Gerontology, Faculty of Medicine, Ain Shams University, Cairo, Egypt; 2 Department of Community Medicine, Environmental, and Occupational Medicine, Faculty of Medicine, Ain Shams University, Cairo, Egypt

**Keywords:** Delirium, Knowledge, Physicians, Education, Attitudes

## Abstract

**Background:**

Delirium is common in older adult population in hospitals. It increases morbidity, mortality, and healthcare cost. Worldwide studies showed that early recognition and proper management are deficient because of knowledge lack among physicians.

**Objective:**

In Egypt, there is a lack of studies assessing delirium knowledge and attitudes in physicians. We aim to assess delirium awareness and attitudes of physicians and pre-registration house officers in Egypt.

**Methods:**

We conducted an online survey evaluating delirium knowledge, diagnostic criteria, and management attitudes. Participants included physicians at various career stages and pre-registration house officers from university and Ministry of Health hospitals across Egypt.

**Results:**

From 1,000 surveys distributed, we obtained 649 responses. Approximately three-quarters of the participants underestimated the prevalence of delirium (77.6%). A few participants knew the outcomes. Knowledge of diagnostic criteria was better. 55.3% of participants use assessment tools as confusion assessment method (CAM). Only about a third had confidence in delirium diagnosis (32.2%) or management (19.4%) and a minority (22.1%) performed serial cognitive assessment in patients at risk of delirium. Only 23.7% reported adequate training. Likely delirium-oriented specialties as geriatrics together with physicians at Ain Shams University hospitals showed more significant knowledge and attitudes to delirium.

**Conclusion:**

We highlighted gaps in delirium awareness and practice among physicians and pre-registration house officers in a variety of hospitals in Egypt. This underlined the need for enhanced delirium education and training.

## Introduction

With the increase in older adult population, delirium, which commonly occurs in older adults, is becoming a significant problem, especially in acute settings. In fact, older adults represent > 40% of hospitalized patients [1]. Delirium is an acute disturbance of cognition and arousal, happening in 10–50% of hospitalized patients. Delirium increases risk of morbidity as dementia, functional decline, institutionalization, and mortality. It increases caregiver burden [2–4] and healthcare cost [5].

Studies have shown that its prevention, early recognition, and management improve outcomes [6]. However, studies also revealed a gap in the practical management of this clinical syndrome [7]. Barriers for following delirium-oriented measures includes lack of physicians' awareness about delirium-associated risks, perceived importance besides disbelief in delirium prevention, lack of confidence in its diagnosis and management [7], or even lack of adherence to evidence-based assessment tools and guidelines to manage it [8–12]. Moreover, delirium treatment mostly depends on the responsible physician’s experience which is mostly pharmacological and junior physicians often prescribe non-specific medications [13]. Also, there is deficient coordination between physicians and nurses [10].

Application of delirium management protocol has been studied across different continents varying from 21.6% in Africa to 90.4% in Australia [13]. However, in Egypt, we do not have a baseline to start with. In Egypt, the problem magnitude is still vague as a few studies detected delirium prevalence [14]. A few studies discussed delirium awareness of healthcare providers, including small samples, mainly nurses, and confines to ICU [15–19]. Therefore, our study aims to assess knowledge and attitudes of physicians, and pre-registration house officer physicians toward delirium. This can be considered a baseline to start future studies. This helps overcoming barriers toward proper delirium care and targeting interventional educational programs.

## Methodology

This was a cross-sectional study involving a survey using a self-administered questionnaire. The questionnaire was constructed by Davis and MacLullich 2009 [6]. Investigators used the questionnaire after taking permission from the original questionnaire authors. It was designed to test knowledge of delirium prevalence, diagnostic criteria using DSM-IV, use of specific screening tools, association with adverse outcomes, and pharmacological management. We assessed attitudes and beliefs related to confidence in delirium diagnosis and its clinical significance using a five-point Likert scale. The survey period was from 17th April 2024 to 11th September 2024. We recruited participants from a convenience sample of physicians and pre-registration house officers. We disseminated a participation request through social media platforms, professional networks, and personal contacts. It included participants from Ain Shams University Hospitals, Azhar University, other university hospitals, and ministry of health hospitals across Egypt. Physician’s career stage involved early career physicians, including pre-registration house officers, general practitioners, and junior residents. Advanced career physicians included senior residents, specialists, and consultants. Those who agreed to participate were invited to complete an online survey and were instructed to complete the response in a single sitting without access to external resources or engaging in discussions. Out of the 1,000 surveys distributed, we received 649 complete responses, 77 invalid responses, yielding a response rate of about 65%. Response rate was high. One reason was the repeated follow-up of requests sent by social media platforms, professional networks, and personal contacts.

## Data management and statistical analysis

Data were tabulated and statistically analysed using SPSS, version 20 (SPSS Inc., Chicago, IL). Qualitative data were expressed as frequencies (*n*) and percentage (%). Chi-square test was used to test the association between qualitative variables. *P* value ≤ 0.05 was considered statistically significant.

## Ethical considerations

Ethical approval was granted by the Ain Shams Faculty of Medicine Research Ethics Committee (Application No. FMASU R73/2024).

This study received approval from Ain Shams Faculty of medicine ethical committee. Approval number is FMASU R73/2024.

- Informed consents were taken directly from participants.
- Confidentiality of participant data was assured.

## Results

Out of the 649 participants, 60.8% were under 30 years old, 61.4% were males, and 61.3% were early career physicians. Regarding the workplace, 42% of participants worked in Ain Shams University hospitals and 57.9% in other hospitals. Specialties with likely delirium orientation as geriatrics, neuropsychiatry, critical care anesthesia, and emergency medicine were only 23.2% (Table 1).

**Table 1:** Demographics participants (total = 649)

	*N* (%)
Age	
< 30 years	395 (60.8%)
30—39 years	124 (19.1%)
40—49 years	111(17.1%)
≥ 50	19 (2.9%)
Gender	
Male	399 (61.4%)
Medical career stage	
Early career	398 (61.3%)
Intermediate or advanced career	251 (38.6%)
Delirium orientation likelihood	
No	498 (76.7%)
Yes	151 (23.2%)
Work or training hospital	
Ain Shams University	273 (42%)
Others*	376 (57.9%)

*N *(%): Number of respondents who answered correctly (percentage of total sample). Others* include: Azhar university, Other University Hospitals, and Ministry of Health Hospitals across Egypt

Only 22.3% of participants correctly identified prevalence of delirium in acutely admitted patients aged over 70. While about 77.7% of them underestimated it. A few participants knew the outcomes of delirium. Additionally, awareness of grave outcomes after delirium was poor, including dementia (18.6%), institutionalization (19.5%), and death (13.2%). This was reflected later in participant’s attitudes regarding their responses to “delirium being distressing for patients”; see Table 3. More than half of physicians correctly identified core delirium diagnostic criteria: inattention (67.8%), acute onset (52.7%), altered arousal (61.4%), and disorganized thinking (73.1%) as core diagnostic features of delirium. However, a large percentage, 47.30% failed to recognize the crucial role of acute onset in delirium diagnosis. Only 19.1% recognized the appropriate dose of haloperidol in severe agitation with delirium (Table 2).

**Table 2:** Distribution of the participants’ correct responses of the delirium knowledge survey

Questionnaire statements	*N* (%)
Knowledge about prevalence	145 (22.3%)
Knowledge about outcomes	
Mortality in delirium associated with UTI	49 (7.5%)
Mortality in delirium with pre-existing cognitive impairment	71 (10.9%)
Relative risk of dementia	121 (18.6%)
Relative risk of institutionalization	127 (19.5%)
Relative risk of death	86 (13.2%)
Knowledge about diagnosis (DSM-IV) and treatment	
Inattention	440 (67.8%)
Acute onset	342 (52.7%)
Altered arousal	250(61.4%)
Disorganized thinking	174 (73.1%)
Appropriate dose of haloperidol in delirium with severe agitation	124 (19.1%)

*N *(%): number of respondents who answered correctly (percentage of total sample). UTI—Urinary Tract Infection. DSM-IV—Diagnostic and Statistical Manual of Mental Disorders, Fourth Edition

Defects in knowledge of delirium prevalence and outcome affected participant’s attitudes. Only about one-third agreed that delirium is underrecognized; about one-third of participants agreed about having a good working knowledge of delirium; and most participants found delirium not distressing for relatives and patients. However, knowledge of diagnostic criteria was better when compared with prevalence and outcome knowledge. Also, 55.3% of participants use validated delirium assessment tools such as CAM. However, this was not adequately reflected on their attitudes toward delirium practice, where only about a third had confidence in delirium diagnosis (32.2%) or management (19.4%) and minority (22.1%) perform serial cognitive assessment in patients at risk of delirium. Moreover, defect in knowledge about treatment was mirrored in that only about a third found delirium to be prevented (31.1%) or treated (30.5%). About half of participants (47.4%) incorrectly agreed that benzodiazepine is a first-line treatment in delirium. About one-fifth was confident in delirium management (19.4%). All the previous results were reflected on their attitudes toward delirium training, as detected in only about a quarter of participants (23.7%) (Table 3).

**Table 3:** Attitudes toward delirium importance

Questionnaire statements	*N* (%)
1. Delirium is underrecognized in acute medical settings	199 (31.1%)
2. Doctors working in acute medical settings should have a good working knowledge of delirium	238 (36.6%)
3. Delirium is distressing for relatives	256 (39.4%)
4. Delirium is distressing for patients	214 (32.9%)
5. I have a good working knowledge of the diagnostic criteria for delirium	209 (32. %)
6. I have used validated delirium assessment tools such as the CAM	359 (55.3%)
7. I routinely undertake serial cognitive assessments in patients at risk of delirium	144 (22.1%)
8. I have had adequate training on delirium	154 (23.7%)
9. I am confident that I can manage Delirium	126 (19.4%)
10. Delirium can be prevented in 30% of inpatients	199 (31.1%)
11. Benzodiazepines are the first-line treatment for delirium	308 (47.4%)
12. I am confident that I can manage Delirium	126 (19.4%)
13. Delirium is a treatable condition	198 (30.5%)

*N *(%): Number of respondents who answered correctly (percentage of total sample). CAM—Confusion Assessment Method

Participants who are likely delirium-oriented significantly recognized prevalence and outcome, which affected their attitudes in recognizing the distress in patients and relatives. Also, likely delirium-oriented participants more significantly recognized some of delirium diagnostic criteria and correct haloperidol dose. This mirrored their attitudes in using CAM significantly, recognizing preventability and disagreeing about benzodiazepines as the first treatment. Moreover, they agreed about the importance of non-pharmacological treatment importance and its limitation because of staff constraints (Table 4).

**Table 4:** Knowledge and attitudes in relation to delirium orientation likelihood

Knowledge of delirium prevalence & outcome	Unlikely delirium oriented	Likely delirium oriented	*P*
	Row %	Row %	
Delirium prevalence	20.6	27.1	0.025*
Delirium is distressing for relatives	36.2	50.9	0.007*
Knowledge about diagnosis (DSM-IV) and treatment			
Inattention	66.8	72.1	0.372
Acute onset	48.4	66.8	< 0.001*
Altered arousal	53.1	66.2	0.024*
Disorganized thinking	71	70.2	0.970
Appropriate dose	14.49	35.10	< 0.001*
Attitudes toward delirium			
I have used validated delirium assessment tools such as the CAM	49.5	75.5	< 0.001*
1. Delirium can be prevented in 30% of inpatients	26.5	45.0	< 0.001*
2. Benzodiazepines are the first-line treatment for delirium	41.8	66.2	< 0.001*
3. Staffing constraints (limitations) often result in the over-use of drug treatments for delirium	24.1	36.4	0.004*

DSM-IV—Diagnostic and Statistical Manual of Mental Disorders, Fourth Edition

CAM—Confusion Assessment Method. Notes: Chi-square test was used

*P value ≤ 0.05 is considered statistically significant

Participants working at Ain Shams University Hospitals were significantly more knowledgeable about prevalence, outcomes, and diagnostic criteria of delirium. This positively affected their attitudes showing confidence in assessment, diagnosis, prevention, and treatment. Furthermore, they were more significantly positive toward delirium training (Table 5).

**Table 5:** Delirium knowledge in relation to working environment

Knowledge of delirium prevalence and outcome	ASU hospitals	Other hospitals	*P*
	Row %	Row %	
Prevalence of delirium and dementia	12.5	6.3	0.012*
Delirium in association with UTI	10.2	5.5	0.023*
Relative risk of dementia	22.4	15.9	0.026*
Knowledge about diagnosis (DSM-IV) and treatment of delirium			
Inattention	76.4	61.2	< 0.001*
Acute onset	61	46.4	0.001*
Altered arousal	60.2	52.5	0.032*
Disorganized thinking	79.4	63.9	< 0.001*
Attitudes toward delirium			
4. I have a good working knowledge of the diagnostic criteria for delirium	36	29.4	0.004*
5. I have had adequate training on delirium	29.7	19.3	0.005*
6. I routinely undertake serial cognitive assessments in patients at risk of delirium	28.3	17.7	< 0.001*
7. Delirium can be prevented in 30% of inpatients	39.7	24.1	< 0.001*
8. Delirium is distressing for patients	44.4	24.6	< 0.001*
9. Delirium is distressing for relatives	51.1	31	< 0.001*
10. I am confident that I can manage delirium	24.6	15.3	0.011*
11. Delirium is a treatable condition	43.3	21.2	< 0.001*
12. Staffing constraints often result in the over-use of drug treatments for delirium	35.6	20.6	< 0.001*

ASU, Ain Shams University Hospitals; UTI—Urinary Tract Infection. DSM-IV—Diagnostic and Statistical Manual of Mental Disorders, Fourth Edition; Notes: Chi-square test was used

*P value ≤ 0.05 is considered statistically significant

## Discussion

The study aims to investigate the knowledge and attitudes toward delirium in physicians in Egypt. In our research, 65% of recruited physicians opted to answer our online delirium survey. Response rate was high. One reason was the repeated follow-up of requests sent by social media platforms, professional networks, and personal contacts.

## Knowledge and attitudes regarding prevalence and outcome

In our study, we found prominent underestimation both in relation to knowledge and attitudes toward delirium prevalence and long-term outcomes. This gap was consistent with the findings of Davis and MacLullich. Although they found that most participants were largely aware of how common and serious delirium is, a notable percentage still underestimated its prevalence. A later study by Jenkin et al. (2016) reported a significant improvement in knowledge, with 82% of participants correctly identifying the prevalence of delirium [6, 20]. However, recognition of the outcomes associated with delirium did not show similar improvement. Additionally, under recognition of delirium was also observed in the two other investigations [21, 22].

## Knowledge and attitudes to diagnostic criteria and management of delirium

We detected good physicians’ knowledge about diagnostic criteria together with the use of validated delirium assessment tools such as CAM. However, this was not reflected on confidence in delirium diagnosis, management. Discrepancy between knowledge and attitudes were similar to some studies which found that although most of participants showed intermediate or advanced knowledge of delirium detection, ED staff correctly identifies delirium only 35% of the time when it is present [23, 24]. Also, Davis & MacLullich, 2009 and Jenkin et al., 2016 demonstrated poor recognition and confidence in diagnosing delirium [6, 20]. Furthermore, Vettasseri et al*.* 2017 reported poor compliance of physicians with guidelines regarding screening for delirium on admission [25]. Additionally, there was a considerable defect in knowledge and attitudes about prevention and treatment. This agreed with findings of Jenkin et al., 2016 [20]. On the other hand, Sinvani et al., 2016 found that the majority of physicians had good knowledge and attitudes toward prevention and treatment [21]. This difference may be attributed to his small sample size.

Although there is good knowledge about diagnosis and assessment, but there is still lack of confidence in ability to manage.

## Effect of delirium orientation likelihood on knowledge and attitudes

Likely delirium-oriented specialties showed better knowledge and attitudes toward delirium practice. This highlights the effect of training through practice on attitudes, including screening and full management of delirium patients. Also, Jenkin et al., 2016 found a significant increase in overall knowledge with geriatricians rather than other specialties like psychiatry and neurology which are possibly also delirium oriented [20]. Chary et al., 2021 reported that delirium prioritization is well characterized with geriatric emergency physicians rather than other specialties [23]. This was in contrast to Davis & MacLullich, 2009 who found that specialty experience (in geriatric medicine) had limited effects on responses [6]. This could be because of changes of training programs across places and time.

## Effect of working environment on knowledge and attitudes

Although our results showed over all defects in physicians training in delirium, there was significantly better knowledge and attitude among the physicians at Ain Shams University (ASU) Hospitals. As a university hospital, there are more opportunities for research and training. At ASU geriatrics hospital, there is undergraduate geriatrics course together with clinical exposure during internship and early career. This provides basic knowledge and clinical competencies related to geriatric syndromes, including delirium. The ASU geriatrics hospital is a stand-alone geriatrics tertiary-care hospital inside ASU hospitals complex. It includes outpatient clinics (primary care geriatrics, general geriatrics, memory clinic, ortho-geriatrics, and palliative medicine); inpatient services (geriatrics wards, intermediate care unit, geriatrics ICU, and palliative medicine unit); and other geriatrics academic and research services. Services are provided by geriatricians and multidisciplinary teams. This facilitate spread of geriatrics knowledge and interactions with physicians through consultations and referrals. Our findings agreed with those of Sinvani et al., 2016 who found that participants who received geriatrics training had significantly better knowledge and more confident in identifying delirium [14]. This was opposite to findings of Chary et al., 2021 who reported the underrepresentation of geriatric emergency medicine content in undergraduate and postgraduate training curricula as well as in licensing examinations [23].

This is the first study in Egypt to assess the awareness and attitudes of physicians to a common geriatrics problem like delirium, especially in acute settings. By enhancing knowledge through educational programs, promoting practical experience, and endorsing evidence-based clinical guidelines, we can improve the recognition and management of delirium, and hence improve both short-term and long-term outcomes on older adult patients and decrease healthcare cost.

## Strengths and limitations

Our strengths included: This is the first Egyptian study to investigate knowledge and attitudes of delirium syndrome among physicians across all Egypt. It included participants from cities from all over Egypt. Covered regions included greater Cairo (20% of population of Egypt), upper Egypt, lower Egypt, and coastal areas. Our study also involved physicians at different career stages and different specialties. It included pre-registration house officers as well. Also, we had high response rate.

However, limitations included: our sample cannot be generalized to represent all Egypt as we used convenient sampling involving those who accepted the online invitation. We could not also get participants from desert regions (5% of the population of Egypt). Our study involves a quantitative nature which does not explore all aspects of deficits in delirium awareness and practice. We recommend performing further future studies.

## Conclusion

Our study probes the current situations regarding delirium knowledge and attitudes among physicians in Egypt. This study highlights the presence of good basics to build upon it, and highlighted the gaps in delirium awareness and practice that are needed to be bridged. In our study, although we found physicians’ underestimation of delirium prevalence and long-term outcomes, we detected good physicians’ knowledge about diagnostic criteria together with the use of validated delirium assessment tools such as CAM. However, this was not reflected on confidence in delirium diagnosis, management. This indicates existence of barriers to confidently translate this knowledge into practice in delirium management. Since the group of “Likely delirium oriented specialties” showed better knowledge and attitudes toward delirium practice, we infer that lack of training is a possible barrier. However, further studies are needed. Significantly better knowledge and attitude among the physicians at Ain Shams University (ASU) Hospitals highlight the importance of encouraging both undergraduate and postgraduate training.
